# Corrélation radio-histologique des lésions mammaires ACR4: à propos de 181 cas et revue de la littérature

**DOI:** 10.11604/pamj.2018.29.140.13699

**Published:** 2018-03-02

**Authors:** Ahmed Guennoun, Yousra Krimou, Chahrazed Bouchikhi, Nisrine Mamouni, Sanaa Errarhay, Abdelaziz Banani

**Affiliations:** 1 Service d’Obstétrique et de Gynécologie I, Hôpital Universitaire Hassan II, Fès, Maroc

**Keywords:** Corrélation radio-histologique, valeur prédictive positive des lésions, sous-catégories ACR4, Radio-histological correlation, positive predictive value of ACR4 lesions, ACR4 sub-categories

## Abstract

La classification Bi-Rads (Breast Imaging Reporting and Data System) de l'ACR (American College of Radiology) est le système de classement des images radiologiques, recommandé pour le dépistage du cancer du sein. La lésion ACR IV correspond à une anomalie indéterminée ou suspecte avec probabilité de malignité de 2-95% selon des études. Cette disparité nous a poussés à réaliser notre étudequi est uneétude rétrospective de 181 patientes étalée sur 5 ans, conduite dans le service de gynécologie-obstétrique I du Centre hospitalier Hassan II de Fès. Notre objectif est de rapporter les résultats histologiques des lésions mammaires classées radiologiquement ACR4 dans le but d'évaluer la corrélation radio-histologique et d'améliorer notre conduite à tenir. Toutes nos patientes ont bénéficié d'une imagerie du sein puis d'une preuve anatomopathologique par différentes techniques. Nous avons noté une nette prédominance des lésions bénignes avec un taux de 62% contre 29% des lésions malignes et seulement 9% étaient intermédiaires. L'adénofibrome est le résultat histologique le plus retrouvé avec 30% des cas, le carcinome canalaire infiltrant reste le chef de file des lésions malignes avec 17% des cas. A travers une revue de la littérature, nos résultats semblent rejoindre ceux des autres études avec une Valeur prédictive positive de 29%. Néanmoins, l'adoption de la sous-classification ACR en ACR4a, b et c parait plus adaptée,en raison du nombre important des interventions chirurgicales inutiles.

## Abstract (en)

The Bi-RADS (Breast Imaging Reporting and Data System) classification developed by the ACR (American College of Radiology) is the classification system for radiological images recommended for breast cancer screening. The ACR 4 microcalcification is an indeterminate or suspected abnormality with 2-95% probability of malignancy, according to studies. This disparity pushed us to conduct this retrospective study of 181 patients in the Department of Obstetrics and Gynecology I at the Hassan II University Hospital, Fez, over a period of 5 years. This study aimed to report the histological results of breast lesions radiologically classified as ACR4 in order to assess their radio-histological correlation and to improve therapeutic approach. All patients underwent breast imaging examinations and then anatomopathologic examination was performed using different techniques. There was a clear predominance of benign lesions with a rate of 62% versus 29% of malignant lesions and only 9% were intermediate lesions. Adenofibroma was the most common histological finding (30% of cases), invasive ductal carcinoma was the most frequent malignant lesion (17% of cases). We performed a literature review which showed that our results were in line with findings of other studies, with a positive predictive value of 29%. Nevertheless, ACR classification divided into subcategories 4a, b and c should be used due to the significant number of unnecessary surgical interventions.

## Introduction

La classification Bi-Rads de l'ACR est le système de classement des images radiologiques, recommandé pour le dépistage du cancer du sein, et permettant de proposer une conduite à tenir adaptée en fonction de ce classement de ACR 1 à ACR 5 selon la probabilité de malignité (Tableau 1). La lésion ACR IV correspond a une anomalie indéterminée ou suspecte avec probabilité de malignité 2-95%. L'attitude diagnostique devant une lésion mammaire ACR4 impose systématiquement une investigation histologique. L'objectif de notre étude est de rapporter les résultats histologiques des lésions mammaires classées radiologiquement ACR4 afin de réaliser une corrélation radio histologique, d'évaluer les résultats retrouvé dans notre service et ainsi améliorer notre conduite à tenir.

**Tableau 1:** Classification ACR

	Signification et conduite à tenir
ACR 0	• Image nécessitant un complément d’imagerie
ACR 1	• Mammographie normale.
ACR 2	• Anomalies bénignes ne nécessitant ni surveillance ni examen complémentaire => Suivi normal (2 ans)
ACR 3	• Anomalie probablement bénigne pour laquelle une surveillance à court terme est conseillée (VPP <2%.) => Surveillance rapprochée tous les 4/ 6 mois pendant 2 ans, si stabilité sur 2 ans et critères bénins = ACR 2.
ACR 4	• **Anomalie indéterminée ou suspecte posant l’indication d’une vérification histologique.** => Prélèvements à visée diagnostique (VPP 2-95%)
ACR 5	• Anomalie évocatrice d’un cancer. => Diagnostic histologique (VPP>95%)
ACR 6	• Anomalie correspondant à un cancer prouvé à l’histologie.

## Méthodes

Il s'agit d'une étude rétrospective étalée sur 5 ans conduite dans le Service de Gynécologie-Obstétrique I du Centre Hospitalier Hassan II de Fès-Maroc. Les patientes de notre série ont été admises pour symptomatologie clinique variée, ou dans le cadre d'une surveillance radiologique programmée, et chez qui nous avons détecté des lésions classées ACR 4. Toutes nos patientes ont bénéficié d'une exploration radiologique: une échographie associée ou non à une mammographie. Nous avons donc complété notre prise en charge par une preuve anatomopathologique par différentes techniques: micro biopsie au trucut simple et sous guidage échographique ou encore chirurgicale avec ou sans repérage radiologique. Nous avons comparé l'aspect radiologique des lésions mammaires classées ACR4 aux résultats histologiques des prélèvements. Les résultats sont présentés sous forme de tableaux et de graphiques, analysés de façon descriptive et corrélationnelle.

## Résultats

Sur les 5ans, l'exploitation des dossiers des patientes ayant des lésion ACR4 a permis de mettre en évidence pour les: données cliniques: l'âge moyen de nos patientes ayant lésions ACR4 est de 40 ans, avec des extrêmes de 13 et 76 ans. 41% de nos patientes étaient nullipares et 24% de nos patientes étaient déjà ménopausées. Pour les antécédents, la prise de contraception orale a été rapportée par 30% des patientes avec une durée moyenne de 3 ans. Au moins 10% des patientes avaientun antécédentpersonnel ou familial de cancer de sein. La palpation d'un nodule était le motif de consultation chez 141 patientes avec une taille moyenne de 2,5 cm. 40 cas ayant bénéficié d'un bilan radiologique sans masse palpable: mastodynies, écoulement, modification cutané ou dans le cadre du dépistage. Nous avons constaté une légère prédominance du sein gauche avec 55,3%, et du QSE avec 19%.

**Données radiologiques:** Toutes nos patientes ont bénéficié d'un examen sénologique: échographie mammaire, mammographie ou IRM mammaire. L'association échographie mammaire et mammographie a été réalisée chez 69% de nos patientes. L'échographie mammaire seule a été indiquée chez des femmes très jeune ou au cours de la grossesse.

**Résultats de la mammographie:** Une opacité a été retrouvée dans 69% des cas, isolée ou associée à des microcalcifications. La mammographie a objectivé des microcalcifications isolées chez 31 patientes, un surcroit de densité chez 14 patientes et une distorsion architecturale chez 7 patientes. Enfin, une mammographie normale a été retrouvée chez 7 patientes soit 6% de cas. Le complément échographique a permis de mettre en évidence la lésion chez ces patientes.

**Résultat de l'échographie:** Une nette prédominance des lésions tissulaires a été retrouvée chez 145 patientes. L'échographie a révélé également une lésion tissulaire endocanalaire (bourgeon endokystique) chez 7 patientes, une dilatation des canaux galactophoriques dans 7 cas. Chez 22 patientes, l'examen a été qualifié comme étant normal,la mammographie a permis de faire le diagnostic chez 17 patientes et l'IRM mammaire chez 5 patientes objectivant un rehaussement sans masse.

**Données histologiques** (Tableau 2): Les lésions malignes représentaient 29% de l'ensemble des lésions recensées dans notre série, avec une prédominance des carcinomes infiltrant (17% des cas). Les adénofibromes constituaient le chef de fils des lésions bénignes avec 30% de l'ensemble des lésions retrouvées, suivie par la mastopathiefibrokystique (12%). Et enfin 9% des lésions étaient intermédiaires représentées par les Tm phyllodes et les Hyperplasie canalaires atypiques.

**Tableau 2:** Résultats histologiques des lésions ACR4

Types histologiques	Nombre de cas	Pourcentage
Lésions malignes	53	**29%**
Carcinome Canalaire Infiltrant	32	17%
Carcinome Canalaire In-situ	6	3%
Association CCI et CCIS	9	5%
Carcinome Lobulaire Infiltrant	2	1%
Carcinome Mucineux	2	1%
Carcinome Myoepithelial	1	1%
Carcinome Micropapillaire Infiltrant	1	1%
Lésions intermédiaires	17	**9%**
Tumeur Phyllode	13	7%
Hyperplasie Canalaire Atypique	4	2%
Lésions bénignes	111	**62%**
Adénofibrome	55	30%
MastopathieFibro-kystique	21	12%
Adénose sclérosante	7	4%
Papillome	5	3%
Hyperplasie Canalaire Simple	4	2%
Lésion de Métaplasie apocrine	3	2%
Lésion de Stéatonecrose	3	2%
Mastite	3	2%
Réaction Inflammatoire	3	2%
Abcès	2	1%
Remaniement Fibreux	2	1%
Kyste	2	1%
Dilatation Galactophorique	1	1%

**Prise en charge thérapeutique:** L'acte chirurgical diagnostique ou thérapeutique a été indiqué chez 82% des patientes et a consisté en une tumorectomie ou mastectomie +/- associées à un curage ganglionnaire. Les indications étaient: les tumeurs malignes et intermédiaires, les adénofibromes de plus de 3cmet en cas de discordance radio-histologique. La surveillance seule a été préconisée seulement chez 18% de nos patientesayant bénéficié d'unemicrobiopsie par trucut avec un résultat anatomo-pathologique en faveur de la bénignité et une concordance radio-histologique.

### Corrélation radio-histologique

**Corrélation écho-histologique** (Tableau 3): l'échographie a permis de mettre en évidence 28% des lésions malignes. L'étude de la valeur P a mis en évidence une différence significative pour les lobulations, les contours et les limites. Ainsi, différentes caractéristiques échographiques ont permis d'établir une description plus précise de la lésion la plus prédictive de malignité: c'est une lésion tissulaire hypo-échogènehétérogène, contenant des microlobulations à contours irréguliers, à limites floues, avec atténuation des échos postérieurs et vascularisée au doppler.

**Tableau 3:** Corrélation echo-histologique

	Nombre		Benin	Malin	P
**Type de lésion**					0,209
Tissulaire	145	90%	68% (99)	32% (46)	
Bourgeon endo-kystique	7	5%	86% (6)	24% (1)	
Dilatation canalaire G	7	5%	86% (6)	14% (1)	
Echogénicite					0,774
Hypoéchogène	125	97%	68% (88)	32% (41)	
Hyperéchogène	4	3%	75% (3)	25% (1)	
Homogénicite					0,22
Homogène	38	31%	82% (31)	18% (7)	
Hétérogène	83	69%	71% (59)	29% (24)	
**Lobulations**					0,007
Absence de lobulation	37	38%	81% (30)	19% (7)	
Lobulations	41	42%	95% (39)	5% (2)	
Microlobulations	19	20%	63% (12)	37% (7)	
**Contours**					0,005
Réguliers	28	38%	79% (22)	21% (6)	
Irréguliers	46	62%	46% (21)	54% (25)	
Limites					0,001
Nettes	28	64%	96% (27)	4% (1)	
Floues	16	36%	56% (9)	44% (7)	
Axe					0,657
Vertical	25	36%	88% (22)	12% (3)	
Horizontal	44	54%	84% (37)	16% (7)	
**Transmission des faisceaux**					0,212
Inchangé	139	78%	73% (102)	27% (37)	
Atténuation	22	12%	59% (13)	41% (9)	
Renforcement	18	10%	83% (15)	17% (3)	
Vascularisation					0,216
Présente	10	71%	60% (6)	40% (4)	
Absente	4	29%	80% (24)	20% (6)	

**Corrélation mammo-histologique** Tableau 4): nous avons trouvé que 36% des patientes ayant bénéficié d'une mammographie avaient un résultat malin. Seules les opacités avaient un résultat significatif (avec une valeur P à 0,001). Par contre pour les micro-calcifications, la distorsion architecturale et le surcroit de densité, les résultats n'avaient pas de valeur significative de malignité.

**Tableau 4:** Corrélation Mammo-histologique

	NOMBRE		BENIN	MALIN	P
**OPACITE**	87	69%	55% (48)	45% (39)	0.001
**TYPE**					0.387
Ronde ou ovale	72	95%	57% (41)	43% (31)	
Lobulée	4	5%	25% (1)	75% (3)	
**CONTOURS**					0.294
Réguliers	20	30%	6% (13)	35% (7)	
Irréguliers	47	70%	51% (24)	49% (23)	
**MICROCALCIFICATION**	32	26%	88% (25)	22% (7)	0.191
**TYPE**					0.959
Rondes	5	16 %	80% (4)	20% (1)	
Punctiformes	11	34 %	82% (9)	18% (2)	
Poussiéreuses	1	3 %	100% (1)	0% (0)	
Polymorphes	15	47 %	73% (11)	27% (4)	
**REGROUPEMENT**					0.197
En Amas	9	82%	88% (8)	12% (1)	
Dispersé	2	18%	50% (1)	50% (1)	
**SURCROIT**	14	11%	71% (10)	29% (4)	0.973
**DISTORSION**	7	6%	57% (4)	43% (3)	0.397

## Discussion

L'hétérogénéité de la catégorie ACR4 et sa VPP qui reste très large a suscité l'intérêt de réaliser une corrélation radio-histologique des lésions retrouvées dans notre étude. D'après notre série et selon une revue de la littérature, aucun signe radiologique n'est spécifique d'un type histologique donné. Ainsi en échographie, devant une lésion bien limitée à contours nets on peut évoquer un fibroadénome mais aussi une tumeur phyllode, un papillome ou un carcinome mucineux. Une masse de contours irréguliers est en général en faveur d'un carcinome infiltrant mais peut être l'aspect d'une cicatrice radiaire ou d'une adénose sclérosante. Nous avons également calculé la VPP de malignité retrouvée dans notre étude qui est de 29%, et nous l'avons comparé aux autres études. Cette valeur était semblable à celle retrouvée dans la majorité des autres études: Orel et al 30%, Gulsun et al 25%, par contre elle était assez disparate dans d'autres études (Tableau 5). Même si notre résultat rejoint la majorité des études, 62% de nos patientes ont bénéficié d'un geste chirurgical par excès (adénofibrome, lésion de stéatonécrose, mastopathiefibro-kystique') (Tableau 2), avec un risque de complication plus important, un retentissement psychique et socialplus marqué, et un cout de prise en charge plus élevée: hospitalisation, anesthésie, bloc et matériels opératoire, médicaments. Une analyse minutieuse des anomalies mammaires est donc indispensable pour relever les caractéristiques suspectes d'une image mammo-échographique.

**Tableau 5:** VPP des lésions ACR4 par rapport aux autres categories

	ACR 3	ACR 4	ACR 5
**ZONDERLAND**	33,9%	52,7%	100%
**OREL**	2%	30%	97%
NOTRE ETUDE		**29%**	
**GULSUN**		25%	68%
**SIEGMANN**	6,3%	16,7%	85%

Ainsi, la 4^ème^ édition du guide BI-RADS a fourni des catégories plus précises du risque de cancer mammaire en créant trois sous-catégories: la catégorie 4A désigne « les lésions avec faible suspicion de malignité. Pour ce groupe, une lésion bénigne à l'histologie est attendue et sera considérée »; La catégorie 4B concerne « les lésions qui ont un niveau de suspicion intermédiaire de malignité. Le suivi et la corrélation histologique sont d'une grande importance dans ce sous groupe car le résultat attendu peut être autant bénin que malin »; La catégorie 4C désigne « les lésions modérément suspectes sans, toutefois des signes classiques de malignité. Le résultat histologique de la malignité est attendu et un résultat concluant à la bénignité sera discordant ». Des études (Tableau 6) [1-7] ont donc été menées afin d'évaluer cette nouvelle sous-classification en comparant la valeur prédictive positive des différentes sous catégories ACR4. Ainsi l'évaluation de la valeur prédictive positive (VPP) pour le cancer du sein dans les études a varié pour les trois sous-groupes: l'ACR4a entre 4 et 10% sauf pour les études de Katarzyn et Rodrigo [1] qui était respectivement à 22 et 17%. L'ACR 4b entre 19 dans l'étude de Torres [5] et 49% et enfin l'ACR 4c entre 52 dans l'étude de Wanaporn [3] et 85%.

**Tableau 6:** VPP des lésions ACR4 et de ses sous-groupes

	ACR 4		ACR 4a	ACR 4b	ACR 4c
	Cas	VPP	VPP	VPP	VPP
Katarzyn	266	52%	22%	49%	70%
Rodrigo Menezes [1]	339	42%	17%	40%	85%
Chaiwerawattana [2]	230	31%	8%	39%	58%
Wanaporn [3]	143	26%	4%	43%	52%
Sanders [4]	191	22%	10%	21%	70%
Torres-Tabanera [5]	880	22%	9%	19%	58%
Cholatip [6]	460	20%	9%	21%	57%
Jung Hyun Yoon [7]	2430	19%	8%	38%	82%

Cette différence de résultat a permis de donner naissance à une 5^ème^ édition de la classification BIRD'S (2013) permettant ainsi d'établir des recommandations du suivi diagnostique et thérapeutique: Catégorie 4A: un suivi à 6 mois après une biopsie bénigne; Catégorie 4B: en fonction de la concordance radio-pathologique précise, un suivi à 6 mois après une biopsie est acceptable pour des atteintes bénignes non prolifératives et prolifératives sans atypie cellulaire; Catégorie 4C: on s'attend à un résultat malin qui devrait justifier une évaluation ultérieure et une biopsie excisionnelle des tous les résultats bénins; La classification BI-RADS4 en trois sous-groupes 4A, 4B, 4C a un intérêt pour: Une meilleure estimation de la valeur prédictive positive de malignité des lésions mammaires; Un suivi diagnostique et une prise en charge thérapeutique plus adaptés; En cas de bénignité, la possibilité de réduire les interventions chirurgicales inutiles et de supprimer les surveillances rapprochées.

## Conclusion

L'analyse pathologique de l'aspect radiologique permet une bonne approche diagnostique toutefois l'examen anatomopathologique reste l'examen fondamental pour déterminer la nature bénigne ou maligne de la tumeur. A travers une revue de la littérature, nos résultats semblent rejoindre ceux des autres études. Cependant le nombre d'intervention chirurgicale inutile reste assez conséquent, la sous-classification ACR en ACR4a, b et c parait une option intéressante pour un meilleur suivi diagnostic, une prise en charge thérapeutique plus adaptée et une surveillance moins rapprochée.

### Etat des connaissances actuelle sur le sujet

- L'hétérogénéité de la catégorie ACR4 et sa VPP qui reste très large de 2 à 95%
- Aucun signe radiologique n'est spécifique d'un type histologique donné;
- La 4^ème^ édition a permis une sous-classification des lésions ACR4 et une prise en charge adaptée pour chaque type.

### Contribution de notre étude à la connaissance

- Rapporter et évaluer l'expérience de notre service dans la prise en charge des lésions ACR 4;
- Décrire et évaluer les lésions échographique et mammographique prédictives de malignité;
- Insister sur l'intérêt de la sous classification dans l'amélioration de la prise en charge des lésions ACR 4.

## Conflits d’intérêts

es auteurs ne déclarent aucun conflit d'intérêts.
